# Efficacy and safety of sintilimab plus a bevacizumab biosimilar combined with transarterial chemoembolization for advanced hepatocellular carcinoma

**DOI:** 10.1097/MD.0000000000046799

**Published:** 2025-12-19

**Authors:** Jiajin Yang, Qiuping Xu, Suo Wu

**Affiliations:** a Department of Oncology, Fengcheng People’s Hospital, Yichun University, Yichun, Jiangxi Province, China.

**Keywords:** bevacizumab, hepatocellular carcinoma, overall survival, sintilimab, transarterial chemoembolization

## Abstract

Sintilimab with a bevacizumab biosimilar has been proved to have promising antitumor activity in patients with unresectable hepatocellular carcinoma (HCC). Patients with advanced HCC also showed promising survival outcomes and substantial response rates with transarterial chemoembolization (TACE). The objective of this study was to investigate the initial clinical effectiveness and safety of combining sintilimab and a bevacizumab biosimilar (Sin + Bev) with TACE in treatment-naive patients with advanced HCC. This retrospective study included patients with advanced HCC who were treated with first-line Sin + Bev and TACE between June 2020 and January 2024. According to modified response evaluation criteria in solid tumors (mRECIST) criteria, we analyzed progression-free survival (PFS), overall survival (OS), and tumor response. Adverse events (AEs) were gathered as well. Cox proportional hazard regression models were used to determine prognostic factors affecting OS and PFS. Twenty-six patients were included. According to mRECIST, the objective response rate was 53.8% (14/26) and the disease control rate was 80.8% (21/26), including 1 patient with complete response (CR). The median follow-up was 28.9 months (IQR, 25.8–32.0). The median PFS and OS were 12.0 months (95% CI: 10.5–13.5) and 23.8 months (95% CI: 19.4–28.2), respectively. Child–Pugh and up-to-seven were both independently correlated with OS. The most frequent AEs included Pyrexia (7 cases) and Decreased neutrophil count (7 cases). Grade 4 AEs occurred in one patient as increased aspartate aminotransferase and alanine aminotransferase, but no 5 AEs were observed. The combination of sintilimab plus a bevacizumab biosimilar with TACE showed a significant therapeutic effect in patients with advanced HCC. Additionally, the AEs associated with this treatment were manageable.

## 1. Introduction

Hepatocellular carcinoma (HCC) is the 6th most common cancer worldwide, the third leading cause of cancer-related death globally, with an estimated 905,677 new cases and 830,180 deaths in 2020.^[1]^ China has the highest prevalence of HCC globally, contributing to almost half of the worldwide HCC burden,^[2,3]^ and the second leading cause of cancer-related death in the region.^[4]^ Despite the improvement of current screening methods, Over 70% of HCC patients are diagnosed with advanced cancer (Barcelona Clinic Liver Cancer (BCLC) stage C) and are not suitable for curative treatments such as resection, transplantation, and ablation.^[5,6]^ These patients have a grim prognosis, with a five-year survival rate of <20%.^[7]^ Since 2007, sorafenib has been shown to significantly prolong the survival of patients with advanced HCC in 2 large randomized clinical trials (SHARP and Oriental study^[8,9]^). Sorafenib was the cornerstone of advanced HCC treatment for ten years until the arrival of lenvatinib in 2018. In the phase III REFLECT trial, lenvatinib significantly improved progression-free survival (PFS) and objective response rate (ORR) according to the modified response evaluation criteria in solid tumors (mRECIST) criteria compared to sorafenib in patients with advanced HCC.^[10]^ However, lenvatinib’s efficacy falls short, with a median overall survival (OS) of 13.6 months.

Inhibitors of programmed death-1 (PD-1) and its ligand (programmed cell death ligand-1) have shown considerable efficacy in advanced HCC. In the phase III HIMALAYA trial^[11]^ and RATIONALE-301,^[12]^ durvalumab and tislelizumab, both PD-(L)1 inhibitors used as monotherapy, have demonstrated non-inferiority to sorafenib in OS, but they have not shown significant improvements in ORR or prolonged OS. Due to the characteristic overactivation of the VEGF pathway in HCC, which may worsen immune evasion, PD-1 and programmed cell death ligand-1 inhibitors have been studied in combination with anti-VEGF agents and multi-kinase inhibitors (MKIs) that have antiangiogenic effects.^[13]^ In the IMbrave150 trial,^[14]^ atezolizumab plus bevacizumab (Atezo + Bev) exhibited significant and clinically meaningful survival benefits over sorafenib in treatment-naive patients with unresectable HCC, achieving a median OS and PFS of 19.2 months (HR: 0.66; 95% CI: 0.52–0.85; *P* = .0009) and 6.9 months (HR: 0.65; 95% CI: 0.53–0.81; *P* < .001), respectively. Similarly, the ORIENT-32 study showed that the anti-PD-1 sintilimab plus a bevacizumab biosimilar (IBI305) (Sin + Bev) exhibited a notable improvement in OS and PFS over sorafenib among Chinese patients.^[15]^ Consequently, they are now established as the primary standard of care for advanced HCC. Nonetheless, in the subset of patients with advanced HCC, the ORIENT-32 trial revealed that the Sin + Bev regimen conferred limited benefits, indicated by an ORR of merely 21% and a median PFS of only 4.6 months. This suggests that the regimen may not yet achieve optimal antitumor response in advanced HCC.

Transarterial chemoembolization (TACE) is the established standard treatment for intermediate-stage HCC worldwide and is frequently utilized in advanced cases as well.^[16]^ TACE induces tumor tissue necrosis and releases tumor antigens, potentially stimulating tumor-specific immune responses.^[17]^ According to the LAUNCH trial,^[18]^ combining TACE with lenvatinib in advanced uHCC patients yielded more favorable outcomes compared to lenvatinib monotherapy. TACE is associated with decreased CD8^+^/PD-1^+^ and CD4^+^/FOXP3^+^, potentially shifting the immunosuppressive microenvironment to an immunosupportive state, thereby augmenting the efficacy of PD-(L)1 inhibitors.^[19]^ Bevacizumab biosimilar (an anti-VEGF antibody) also has a synergistic antitumor effect by inhibiting the overexpression of VEGF induced by TACE. This offers the basis for considering a potential synergistic anti-tumor effect through the combination of TACE with sintilimab and a bevacizumab biosimilar for advanced HCC.

Hence, we propose that combining Sin + Bev with TACE could offer a promising and effective therapeutic avenue for advanced HCC patients, potentially informing multidisciplinary precision treatments for HCC. As there is currently no research on the combination of Sin + Bev with TACE in advanced HCC treatment, this retrospective study aims to assess its safety and initial antitumor efficacy.

## 2. Patients and methods

### 2.1. Patients

This retrospective study included advanced HCC patients who underwent treatment with the combination of sintilimab (S) and a bevacizumab biosimilar (B) alongside TACE (T) (sintilimab combined with bevacizumab biosimilar and transarterial chemoembolization [SBT]) at the Oncology Department of Fengcheng People’s Hospital in Jiangxi Province, China between June 2020 and January 2024. We enrolled individuals who fulfilled the following criteria: aged 18 years or older, HCC was radiologically or histologically confirmed according to American Association for the Study of Liver Disease criteria [AASLD guidelines],^[20]^ There is at least one measurable lesion based on the mRECIST (version 1.1),^[21]^ Patients with advanced HCC were diagnosed at the BCLC stage C according to clinical guidelines,^[22]^ Eastern Cooperative Oncology Group (ECOG) Performance score of 0 to 2, Child–Pugh (CP) grade A/B, and Administration of Sin + Bev within 3 weeks following TACE. Criteria for exclusion were: concomitant with other malignant neoplasms, use of systemic therapy other than sintilimab and bevacizumab biosimilar, multiple organ failure, and missing clinical data and loss to follow up. This research scheme has been approved by the Ethics Committee of Fengcheng People’s Hospital of Jiangxi Province, China. All the participants in the manuscript provided written informed consent.

### 2.2. TACE procedure

Interventional radiologists, each with over a decade of experience, conducted the TACE procedures. They administered local anesthesia as part of the treatment process. Employing the Seldinger method, they punctured the femoral artery, utilized digital subtraction angiography to identify tumor-feeding arteries, and subsequently inserted the catheter into these arteries. Chemoembolization was performed using a mixture of lipiodol and pirarubicin (25–40 mg/m^2^). The necessity for repeated TACE was assessed based on enhanced CT or MR results and tumor markers, using an “on-demand” approach.

### 2.3. Sintilimab + bevacizumab biosimilar

Based on the patient’s status, intravenous administration of sintilimab and a bevacizumab biosimilar was initiated within 3 weeks post-TACE. Sintilimab was administered at the standard dose of 200 mg per infusion, followed by intravenous administration of bevacizumab at a dosage of 15 mg/kg. This treatment schedule was repeated every 3 weeks until disease progression or unacceptable adverse effects occurred.

### 2.4. Data collection and follow-up

All baseline data, including laboratory and imaging data, were recorded before the triple combination treatment, including sex, age, cirrhosis, Eastern Cooperative Oncology Group Performance Status score, hepatitis, α-fetoprotein level, CP class, BCLC, with or without macrovascular invasion and/or extrahepatic metastasis, up-to-seven, Esophageal varices, number of TACE sessions. The primary endpoints of this study were PFS and ORR. PFS was defined as the duration from the initiation of the triple combination treatment to either disease progression or death from any cause. Tumor responses were classified based on mRECIST and RECIST version 1.1 criteria, including progressive disease (PD), stable disease (SD), partial response (PR), and complete response (CR). ORR was the proportion of patients achieving a CR and PR. Secondary study endpoints included OS, disease control rate (DCR) and adverse events (AEs). OS was the time from the start of the triple combination treatment to death from any cause, DCR referred to the sum of PR, SD, and CR. Treatment-related adverse events (TRAEs) were graded using the National Cancer Institute General Toxicity 5.0 criteria.^[23]^ Data collection was conducted through outpatient visits, medical records, or telephone follow-up. The deadline for follow-up is January 2024. Tumor response was assessed by contrast-enhanced MR or CT every 4 to 8 weeks, and the patient’s follow-up data were recorded every 2 months until the disease progression or death.

## 3. Statistical analysis

Continuous variables with normal or non-normal distributions were expressed as mean ± standard deviation (SD) or median (range), which were analyzed using Student *t* test. Categorical variables were analyzed using the χ^2^ test, and were described in terms of frequency and proportion. PFS and OS were calculated using the Kaplan–Meier method, with A log-rank test between subgroups. Factors with *P* < .1 in univariate analysis were subsequently included in a Cox proportional hazards regression model to identify factors independently associated with PFS. A significance level of *P* < .05 was considered statistically significant. All statistical analyses were conducted using SPSS 27.0 software (IBM Corporation, Armonk).

## 4. Result

### 4.1. Patient characteristics

Between June 2020 and January 2024, a total of 26 patients were included in the study (Table 1). All patients were infected with hepatitis B virus and received the SBT regimen. The median age of the patients was 57.5 years (range 39–78), with 22 (84.6%) being male. The median number of TACE in SBT group was one time (interquartile range (IQR), 1–2 cycles), 15 patients (57.7%) received one time TACE and only 2 (7.7%) patients received 4 times TACE treatments. Among the patients, 20 (76.9%) had an ECOG performance status of 0 to 1, 18 (69.2%) had CP grade A, 20 (76.9%) had extrahepatic metastases, 12 (46.2%) had microvascular invasion, 7 (26.9%) had liver cirrhosis and All patients had BCLC stage C. There were 7 (26.9%) patients with mild esophageal varices and 19 (73.1%) patients without esophageal varices.

**Table 1:** Characteristics of patients in this study.

Characteristic	No. (%)
Age (yr)	
≤50	8 (30.8%)
>50	18 (69.2%)
Gender	
Female	4 (15.4%)
Male	22 (84.6%)
HBV infection	26 (100.0%)
Liver cirrhosis	
No	19 (73.1%)
Yes	7 (26.9%)
Child–pugh class	
A	18 (69.2%)
B	8 (30.8%)
AFP (ng/mL)	
≤400	16 (61.5%)
>400	10 (38.5%)
ECOG- PS	
0–1	20 (76.9%)
2	6 (23.1%)
BCLC stage C	26 (100.0%)
Microvascular invasion	
No	14 (53.8%)
Yse	12 (46.2%)
Extrahepatic metastasis	
No	6 (23.1%)
Yes	20 (76.9%)
Up-to-seven	
≤7	9 (34.6%)
>7	17 (65.4%)
Esophageal varices	
None	19 (73.1%)
G1 (mild)	7 (26.9%)
No. of TACE	
1–2	21 (80.8%)
≥3	5 (19.2%)

AFP = α-fetoprotein, BCLC = Barcelona clinic liver cancer, ECOG PS = Eastern cooperative oncology group performance status, HBV = hepatitis B virus, TACE = transarterial chemoembolization.

### 4.2. Efficacy

**Figure 1.**
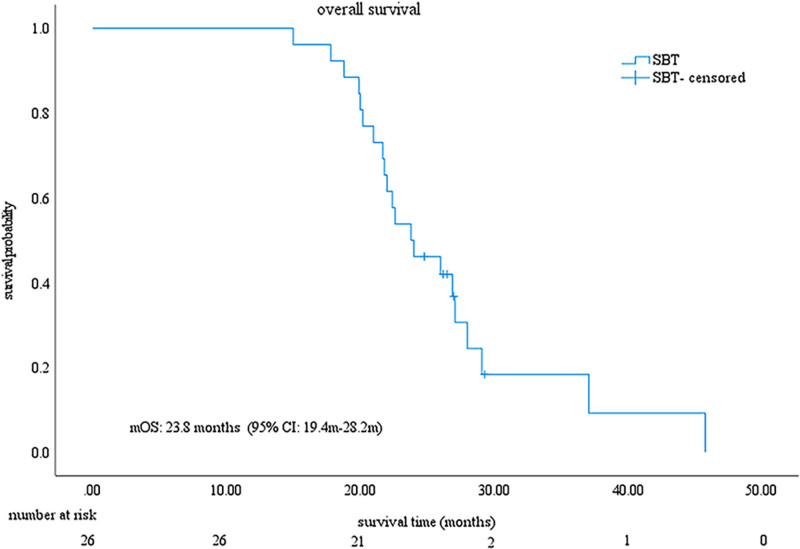
Kaplan–Meier curve of OS for patients in this study. The mOS was 23.8 months (95% CI: 19.4–28.2 mo). The solid line represents the survival curve for the SBT group; crosses indicate censored observations. The x-axis shows survival time in months. CI = confidence interval, mOS = median overall survival, SBT = sintilimab combined with bevacizumab biosimilar and transarterial chemoembolization.

**Figure 2.**
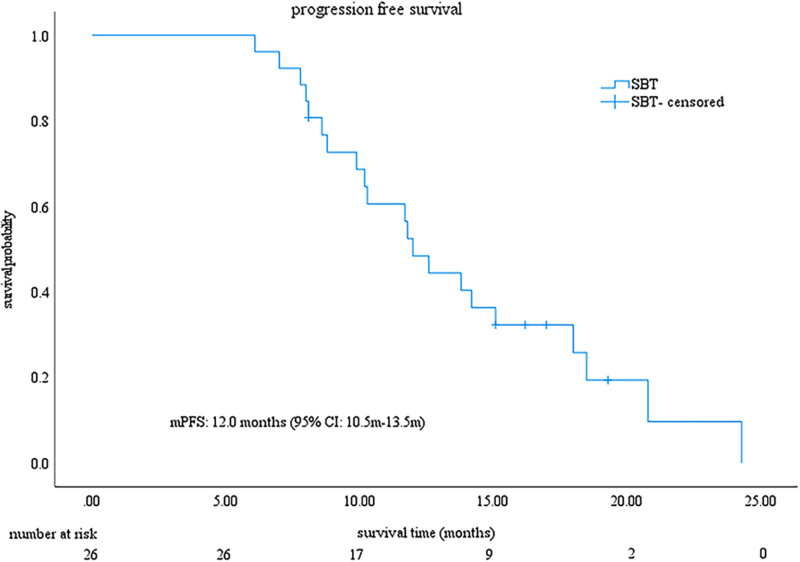
Kaplan–Meier curve of PFS for patients in this study. The mPFS was 12.0 months (95% CI: 10.5–13.5 mo). The solid line represents the PFS curve for the SBT group; crosses indicate censored observations. The x-axis shows time in months. CI = confidence interval, mPFS = median progression-free survival, SBT = sintilimab combined with bevacizumab biosimilar and transarterial chemoembolization.

**Figure 3.**
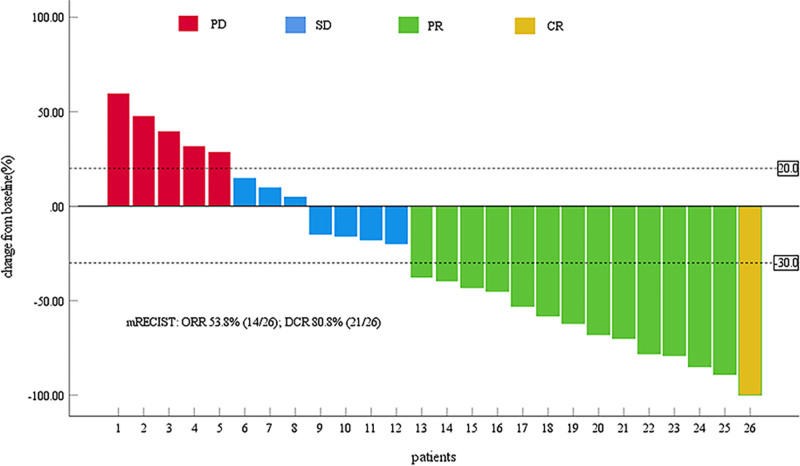
Tumor response evaluation based on mRECIST criteria. Waterfall plot showing the maximum percentage change in tumor size from baseline for each patient. Bar chart showing the distribution of best overall response categories, with colors representing different response types: CR (yellow), PR (green), SD (blue), and PD (red). According to mRECIST, the ORR was 53.8% (14/26) and the DCR was 80.8% (21/26). CR = complete response, DCR = disease control rate, mRECIST = modified response evaluation criteria in solid tumors, ORR = objective response rate, PD = progressive disease, PR = partial response, SD = stable disease.

As of March 1, 2024, the median follow-up duration was 28.9 months (IQR, 25.8–32.0). Throughout the follow-up period, 21 patients (80.8%) had died. The median PFS and OS were 12.0 months (95% CI: 10.5–13.5), and 23.8 months (95% CI: 19.4–28.2), respectively (Figs. 1 and 2). Based on mRECIST criteria, the ORR was 53.8% (14/26), with a DCR of 80.8% (21/26), including one patient achieving CR, 13 with PR, 7 with SD, and five with PD. According to RECIST1.1, the ORR was 46.2% (12/26), with a DCR of 80.8% (21/26), comprising 12 with PR, 9 with SD, and 5 with PD, with no patient with CR (Table 2). The change in target lesion in 26 patients, as per mRECIST criteria, was assessed relative to the baseline (Fig. 3).

**Table 2:** Response to treatment according to mRECIST and RECIST1.

Tumor response	RECIST1.1 n (%)	mRECIST n (%)
CR	0	1 (3.8%)
PR	12 (46.2%)	13 (50.0%)
SD	9 (34.6%)	7 (26.9%)
PD	5 (19.2%)	5 (19.2%)
ORR (CR + PR)	12 (46.2%)	14 (53.8%)
DCR (CR + PR + SD)	21 (80.8%)	21 (80.8%)

CR = complete response, DCR = disease control rate, mRECIST = modified response evaluation criteria in solid tumors, ORR = objective response rate, PD = progressive disease, PR = partial response, SD = stable disease.

**Figure 4.**
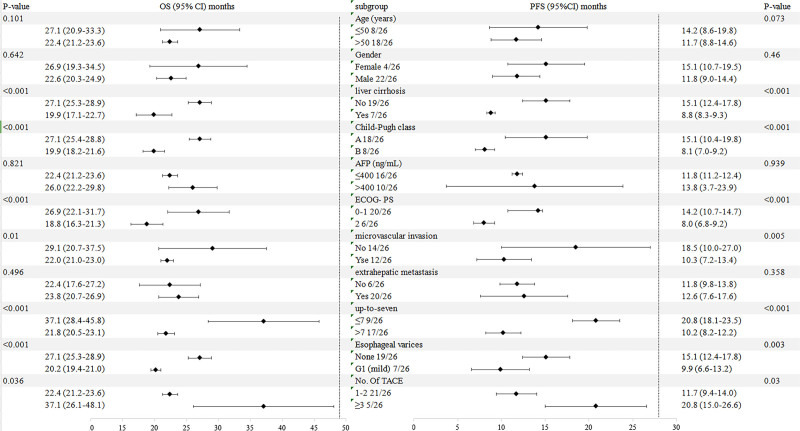
Subgroup analysis of PFS and OS according to baseline characteristics. The median OS and PFS (in months) with 95% CIs are provided for each category. *P*-values indicate the significance of differences between subgroups. AFP = α-fetoprotein, CI = confidence interval, ECOG-PS = Eastern cooperative oncology group performance status, OS = overall survival, PFS = progression-free survival, TACE = transarterial chemoembolization.

Figure 4 depicts the analysis of OS and PFS across various subgroups. Poisson regression with robust error variance was used to estimate treatment effectiveness and 95% confidence intervals. Consistent benefits in OS and PFS were observed across subgroups with ECOG-PS 0 to 1, no liver cirrhosis, no microvascular invasion, up-to-seven ≤ 7, none Esophageal varices and 1 to 2 times TACE reatments. Both PFS and OS showed no statistically significant differences across subgroups stratified by α-fetoprotein levels, age, gender, or extrahepatic metastasis.

### 4.3. Prognostic factors affecting PFS and OS

Table 3 shows the results of univariate analysis for PFS and OS, while Table 4 presents the results of multivariate analysis for PFS and OS. The univariate analysis showed that PFS and OS were influenced by liver cirrhosis, CP, ECOG-PS, microvascular invasion, up-to-seven, Esophageal varices and numble of TACE. Multivariate Cox regression analysis revealed that CP and up-to-seven were independently associated with OS.

**Table 3:** Univariate and multivariate analysis of risk factors for progression-free survival.

Variable	Univariate analysis			Multivariate analysis		
	HR	95% CI	*P*-value	HR	95% CI	*P*-value
Age (≤50 vs >50)	2.505	0.890–7.050	.082	3.207	0.859–11.971	.083
Gender (Female vs Male)	1.589	0.460–5.489	.464			
Liver cirrhosis (no vs yes)	6.339	1.947–20.636	.002	0.115	0.005–2.523	.170
Child–Pugh (A vs B)	8.922	2.582–30.828	<.001	15.93	0.985–257.718	.051
ECOG PS (0–1 vs 2)	9.326	2.516–34.576	<.001	1.826	0.372–8.951	.458
AFP (ng/mL) (≤400 vs >400)	0.966	0.393–2.371	.939			
Microvascular invasion (no vs yes)	3.941	1.431–10.854	.008	3.844	0.817–18.085	.088
Extrahepatic metastasis (no vs yes)	0.632	0.235–1.698	.363			
Up-to-seven (≤7 vs >7)	24.482	3.057–196.033	.003	7.580	0.661–86.942	.104
Esophageal varices (none vs G1)	4.532	1.544–13.308	.006	2.468	0.592–10.288	.215
No. of TACE	0.221	0.050–0.975	.046	0.331	0.039–2.807	.311

AFP = α-fetoprotein, CI = confidence interval, ECOG PS = Eastern cooperative oncology group performance status, HR = hazard ratio, TACE = transarterial chemoembolization.

**Table 4:** Univariate and multivariate analysis of risk factors for overall survival.

Variable	Univariate analysis			Multivariate analysis		
	HR	95% CI	*P*-value	HR	95% CI	*P*-value
Age (≤50 vs >50)	2.343	0.826–6.645	.109			
Gender (Female vs Male)	1.339	0.389–4.611	.643			
liver cirrhosis (no vs yes)	10.794	3.029–38.467	<.001	1.416	0.129–15.543	.776
Child–Pugh (A vs B)	44.561	5.304–374.367	<.001	13.255	1.143–153.742	.039
ECOG PS (0–1 vs 2)	6.335	2.181–18.396	<.001	2.830	0.746–10.741	.126
AFP (ng/mL) (≤400 vs >400)	0.902	0.367–2.215	.821			
microvascular invasion (no vs yes)	3.413	1.262–9.225	.016	0.726	0.165–3.204	.673
extrahepatic metastasis (no vs yes)	0.712	0.266–1.905	.498			
up-to-seven (≤7 vs >7)	25.906	3.265–205.558	.002	19.588	1.362–281.699	.029
Esophageal varices (none vs G1)	7.08	2.192–22.871	.001	3.018	0.640–14.230	.163
No. of TACE	0.229	0.052–1.016	.053	1.026	0.151–6.964	.979

AFP = α-fetoprotein, CI = confidence interval, ECOG PS = Eastern cooperative oncology group performance status, HR = hazard ratio, TACE = transarterial chemoembolization.

### 4.4. Safety

Common AEs are summarized in Table 5. All 26 patients (100%) who underwent SBT therapy experienced at least one TRAE. The most frequent AEs were Pyrexia (7 cases), Decreased neutrophil count (7 cases), fatigue (6 cases), nausea (5 cases), hypertension (5 cases). Grade 3 to 4 adverse reactions occurred in 15.4% of cases, include Decreased neutrophil count (1 case), Gastrointestinal hemorrhage (1 case) and liver failure (1 case), No treatment-related death occurred in the SBT group. Sintilimab was discontinued due to increased AST and ALT in 1 (3.8%) patients. bevacizumab biosimilar was discontinued in 1 (3.8%) patients because of Gastrointestinal hemorrhage. Symptoms or signs of related AEs were alleviated or resolved following symptomatic treatment, dosage adjustment, or temporary cessation of medication.

**Table 5:** Summary of adverse events.

Adverse events n (%)	SBT group (n = 26)				
	Any grade	Grade 1	Grade 2	Grade 3	Grade 4
TRAEs	71 (100%)	56 (100%)	11 (42.3%)	2 (7.7%)	2 (7.7%)
Increased AST	4 (15.4%)	3 (11.5%)	0	0	1 (3.8%)
Increased ALT	3 (11.5%)	2 (7.7%)	0	0	1 (3.8%)
Hypertension	5 (19.2%)	5 (19.2%)	0	0	0
Diarrhea	3 (11.5%)	2 (7.7%)	1 (3.8%)	0	0
Proteinuria	3 (11.5%)	2 (7.7%)	1 (3.8%)	0	0
Hypothyroidism	4 (15.4%)	4 (15.4%)	0	0	0
Pyrexia	7 (26.9%)	6 (23.1%)	1 (3.8%)	0	0
Hyperbilirubinemia	2 (7.7%)	2 (7.7%)	0	0	0
Fatigue	6 (23.1%)	5 (19.2%)	1 (3.8%)	0	0
Weight loss	2 (7.7%)	2 (7.7%)	0	0	0
Decreased appetite	4 (15.4%)	3 (11.5%)	1 (3.8%)	0	0
Decreased neutrophil count	7 (26.9%)	4 (15.4%)	2 (7.7%)	1 (3.8%)	0
Decreased platelet count	3 (11.5%)	3 (11.5%)	0	0	0
Abdominal pain	3 (11.5%)	3 (11.5%)	0	0	0
Abdominal distension	4 (15.4%)	3 (11.5%)	1 (3.8%)	0	0
Nausea	5 (19.2%)	4 (15.4%)	1 (3.8%)	0	0
Vomiting	4 (15.4%)	3 (11.5%)	1 (3.8%)	0	0
Gastrointestinal haemorrhage	1 (3.8%)	0	0	1 (3.8%)	0
Ascites	1 (3.8%)	0	1 (3.8%)	0	0

ALT = alanine aminotransferase, AST = aspartate aminotransferase, SBT = sintilimab combined with bevacizumab biosimilar and transarterial chemoembolization, TRAE = treatment-related adverse event.

## 5. Discussion

The LAUNCH trial revealed that lenvatinib plus TACE led to enhanced OS in contrast to lenvatinib monotherapy.^[18]^ This outcome is particularly promising given that previous studies combining TACE with targeted agents such as sorafenib,^[24]^ brivanib,^[25]^ and orantinib,^[26]^ failed to demonstrate a survival benefit. To our knowledge, this study marks the first exploration documenting the efficacy and safety of sintilimab combined with a bevacizumab biosimilar alongside TACE as an initial treatment for advanced HCC. Following stringent inclusion criteria, the initial findings of this retrospective analysis indicate that the triple therapy comprising Sin + Bev in conjunction with TACE yielded a substantial ORR (53.8%) based on mRECIST criteria, along with a median PFS of 12.0 months in patients with treatment-naive advanced HCC, demonstrating promising efficacy and manageable safety profiles.

In this study, most patients had high tumor burden (65.4%), extrahepatic metastasis (76.9%), or CP class B (30.8%), and These patients were perceived to have a markedly low likelihood of survival benefit. Nonetheless, our study showed that SBT significantly improved the endpoints of patients with advanced HCC, demonstrating elevated response rates and improved survival outcomes. In advanced HCC, The triple therapy demonstrated superior ORR and PFS compared to previous studies involving atezolizumab plus bevacizumab,^[14]^ sintilimab plus bevacizumab biosimilar,^[15]^ camrelizumab–rivoceranib,^[27]^ tremelimumab plus durvalumab,^[11]^ and other TKI therapies.^[8,9]^ Previous studies reported ORRs ranging from 2 to 33.2% and PFS durations of 2.8 to 6.8 months. Notably, we observed that one patients (3.8%) achieved CR, which suggested that could achieve longer survival times. TACE may modulate the immunosuppressive tumor microenvironment by reducing immunosuppressive cell subsets (including PD-1⁺ T cells and monocytes),^[28–30]^ thereby potentiating the antitumor effects of PD-1 inhibitors such as sintilimab. The multivariate analysis of this study showed that CP and up-to-seven are independent risk factors for OS, consistent with previous studies.^[31]^ Henceforth, it is imperative to prioritize investigations into the immunosuppressive microenvironment and elucidate the mechanisms of drug resistance in HCC, particularly in relation to CP classification and tumor burden. Immunomodulatory processes within the tumor microenvironment (TME), involving T cells, macrophages, and myeloid-derived suppressor cells, play a pivotal role in determining HCC outcomes by modulating tumor proliferation, immune evasion, and therapeutic response.^[32,33]^ In addition, recent reviews have largely concentrated on the biological uses and therapeutic potential of the most important sirtuins inhibitors and activators for cancer and other diseases, including HCC.^[34–36]^

The efficacy of combining therapies with distinct mechanisms has been validated in the treatment of advance HCC.^[37,38]^ While TACE is a standard local therapy for intermediate or advanced unresectable HCC, challenges such as persistent residual tumors and increased recurrence rates significantly affect the long-term survival of patients with advanced HCC.^[39]^ Sintilimab combined with a bevacizumab biosimilar has the potential to alter the hypoxic tumor microenvironment, which caused by TACE. TACE also can enhance the antitumor effect of Immune Checkpoint Inhibitors by reduce tumor burden and releasing tumor antigens.^[17,19]^ Hence, the triple therapy comprising sintilimab, a bevacizumab biosimilar, and TACE holds promise for swiftly diminishing tumor burden, extending the duration of systemic therapy response, and ultimately enhancing long-term patient survival. In this study, the use of TACE to reduce tumor burden, followed by Sin + Bev as systemic therapy, led to a median PFS of 12.0 months, which exceeded the 4.6 months observed in the ORIENT-32 trial.^[15]^ This implies a potential limitation in the clinical efficacy of Sin + Bev in advanced HCCs, with TACE potentially enhancing overall efficacy. Since the cohort is restricted to hepatitis B virus related HCC, the treatment responses would be interpreted cautiously in the case of conditions such as hepatitis C virus-related and nonalcoholic fatty liver disease-related HCC. Further multi-center studies involving more diverse etiologies are warranted to validate the generalizability of these findings across the broader HCC population. Notably, other promising immunotherapeutic strategies, such as chimeric antigen receptor T-cell therapy, have demonstrated efficacy in treating a variety of solid tumors (e.g., HCC, Prostate Cancer).^[40,41]^

TRAEs were relatively common with triple therapy in this study, but there were no deaths attributed to AEs. Although grade 3 to 4 Decreased neutrophil count and elevated aspartate aminotransferase/alanine aminotransferase (ALT) caused by SBT therapy were observed, they could be managed through dose adjustments and discontinuation of treatment when necessary. The most commonly associated AEs were Pyrexia (7 cases), Decreased neutrophil count (7 cases), fatigue (6 cases), nausea (5 cases), hypertension (5 cases), consistent with the previous study. According to previous research, Sin + Bev May result in patient death due to Gastrointestinal hemorrhage. So we enrolled patients with advanced HCC with no/mild esophageal varices. However, 1 (3.8%) patient had gastrointestinal hemorrhage that led to discontinuation of bevacizumab biosimilar. 1 (3.8%) patient developed immune hepatitis with elevated ALT and aspartate aminotransferase, leading to discontinuation of sintilimab. TACE-related TRAEs, such as nausea, abdominal pain and vomiting, were in line with findings reported in previous studies.^[42,43]^ We did not detect any additional potential AEs in this study, thereby demonstrating the clinical feasibility and safety of triple therapy.

While our study presented initial clinical findings of Sin + Bev combined with TACE for advanced HCC, offering clinical insights for future trials, several limitations should be acknowledged. First, as a retrospective study conducted at a single institution, the findings may be subject to selection bias. Second, the inherent limitations of the retrospective design, combined with the relatively small sample size, should be taken into account when interpreting the results. Third, performing TACE according to a standardized procedure remains challenging, even with 2 experienced interventional physicians, as ensuring consistent assurance of embolism degree and drug dosage is difficult. Furthermore, as this study exclusively enrolled treatment-naïve patients with advanced HCC, caution must be exercised in generalizing these findings to all HCC patients. Therefore, larger, multi-center cohort studies with long-term follow-up are needed to verify the present results and to identify independent influencing factors of this combination therapy.

In conclusion, the combination of Sin + Bev with TACE demonstrated significant therapeutic efficacy and manageable AEs in treatment-naive patients with advanced HCC. This triple therapy regimen involving sintilimab, a bevacizumab biosimilar, and TACE holds promise as a potential alternative treatment for HCC. The notable ORR observed with this triple-therapy regimen holds transformative potential for the management of patients with locally advanced HCC.

## Author contributions

**Conceptualization:** Suo Wu.

**Data curation:** Jiajin Yang, Qiuping Xu, Suo Wu.

**Formal analysis:** Jiajin Yang, Qiuping Xu, Suo Wu.

**Funding acquisition:** Jiajin Yang, Qiuping Xu, Suo Wu.

**Investigation:** Jiajin Yang, Qiuping Xu, Suo Wu.

**Methodology:** Jiajin Yang, Suo Wu.

**Project administration:** Jiajin Yang, Suo Wu.

**Resources:** Jiajin Yang, Suo Wu.

**Software:** Jiajin Yang, Qiuping Xu, Suo Wu.

**Supervision:** Jiajin Yang, Suo Wu.

**Validation:** Jiajin Yang, Qiuping Xu, Suo Wu.

**Visualization:** Jiajin Yang, Suo Wu.

**Writing – original draft:** Jiajin Yang, Suo Wu.

**Writing – review & editing:** Jiajin Yang, Suo Wu.
